# Regulatory role and mechanism of sRNA24 targeting pqsH in *Pseudomonas aeruginosa* infection immunity

**DOI:** 10.3389/fcimb.2026.1933779

**Published:** 2026-10-08

**Authors:** Huanlan Liao, Qianying Liu, Yuyang Liu, Qiwei Li, Yujuan Xiong, Lan Wang, Yang Lu

**Affiliations:** 1 Department of Laboratory Medicine, The Second Affiliated Hospital of Guangzhou University of Chinese Medicine, Guangzhou, Guangdong, China; 2 Guangzhou University of Chinese Medicine, Guangzhou, Guangdong, China; 3 Department of Laboratory Medicine, Panyu Hospital of Guangzhou University of Chinese Medicine, Guangzhou, Guangdong, China; 4 Department of Laboratory Medicine, Chengdu Integrated TCM & Western Medicine Hospital, Chengdu, Sichuan, China; 5 Department of Clinical Laboratory, The Affiliated Qingyuan Hospital of Guangzhou Medical University (Qingyuan People’s Hospital), Qingyuan, Guangdong, China

**Keywords:** immune evasion, pqsH, *Pseudomonas aeruginosa*, quorum sensing, small RNA, virulence regulation

## Abstract

**Introduction:**

*Pseudomonas aeruginosa* is an opportunistic pathogen that causes healthcareassociated infections through complex virulence mechanisms and immune evasion strategies. The quorumsensing (QS) system is central to its pathogenesis, whereas small noncoding RNAs (sRNAs) modulate bacterial adaptability and virulence. This study investigates the regulatory function of sRNA24 during infection.

**Methods:**

This study used a mouse intraperitoneal infection model and RNA sequencing for screening to identify and explore the regulatory function of sRNA24 during infection. Bacterial proliferation, virulencerelated phenotypes, host cell adhesion/invasion, and immunerelated transcriptional levels were subsequently assessed. Mechanistic studies focused on the functional targeting of pqsH, a crucial QS synthase gene. Results:The expression of sRNA24 is dynamically regulated by the host immunological microenvironment. sRNA24 does not influence bacterial proliferation but promotes pyocyanin synthesis, biofilm development, and host cell adhesion/invasion, while inhibiting swimming/swarming motility. Immunologically, sRNA24 suppresses M1 macrophage markers and pro-inflammatory cytokines, promotes M2 markers and antiinflammatory cytokines, and exhibits an anti-inflammatory-biased transcriptional profile. Mechanistically, sRNA24 functionally targets and upregulates pqsH expression. Overexpression of pqsH mimics the phenotypes induced by sRNA24 overexpression. In vivo, the sRNA24-overexpressing strain exhibits enhanced peritoneal colonization.

**Discussion:**

These findings reveal a previously unrecognized sRNAmediated regulatory axis in *P. aeruginosa* infection, suggesting sRNA24 as a potential target for further investigation in the context of persistent infections.

## 1 Introduction

*Pseudomonas aeruginosa* is an opportunistic pathogen and a major cause of healthcare-associated infections, especially acute and chronic infections in immunocompromised patients, burn victims, and individuals with cystic fibrosis (Millar et al., 2018). The treatment of *P. aeruginosa* infections encounters significant challenges, primarily due to its antibiotic resistance and acquired resistance mechanisms, which promote adaptive mutations during prolonged antibiotic therapy, resulting in the development of multidrug-resistant strains. The World Health Organization has classified carbapenem-resistant *P. aeruginosa* as a high-priority bacterium, highlighting the urgent need for novel treatment approaches (Sati et. al. 2024).

Beyond resistance, *P. aeruginosa* employs a sophisticated virulence regulatory network, with the quorum sensing (QS) system controlling biofilm formation, pyocyanin production, and toxin secretion (Schuster and Greenberg, 2006). Among post-transcriptional regulators, small non-coding RNAs (sRNAs) have emerged as key modulators of bacterial adaptation and pathogenicity in many pathogens, including *Staphylococcus aureus* and *Salmonella* (Gottesman and Storz, 2011). However, in *P. aeruginosa*, although several sRNAs [e.g., PhrS (Sonnleitner et al., 2011), PrrF (Djapgne et al., 2018), RsmZ (Jia et al., 2023)] are known to regulate virulence factors, a critical knowledge gap remains: how sRNAs function as upstream hubs to both sense the host immune environment and actively manipulate host immunity to facilitate chronic infection is largely unexplored.

Extensive studies demonstrate that sRNA-mediated regulation of pathogen virulence is a widespread and essential phenomenon (Brencic et al., 2009; Duss et al., 2014). In numerous diseases, sRNAs precisely regulate the expression of virulence factors through diverse mechanisms. For instance, *Staphylococcus aureus* RNAIII targets spa mRNA and inhibits the Rot transcription factor, thereby upregulating hemolysin and protease expression to facilitate bacterial invasion (Boisset et al., 2007); *Staphylococcus epidermidis* RsaE targets icaR mRNA, thereby promoting polysaccharide intercellular adhesin (PIA) synthesis and enhancing biofilm formation (Schoenfelder et al., 2019). *Salmonella* InvR and SdsR regulate outer membrane proteins and flagellar genes, respectively, optimizing host cell invasion and intracellular survival (Pfeiffer et al., 2007; Monteiro et al., 2012). Collectively, these studies illustrate a complex and sophisticated sRNA-mediated virulence regulatory network.In *P. aeruginosa*, sRNAs hypoxia induced PhrS activates PqsR, upregulating PQS synthesis and pyocyanin production (Sonnleitner et al., 2011). under iron limitation, PrrF1/F2 modulate PQS synthase genes, enhancing virulence factor output (Reinhart et al., 2017). RsmA acts as a positive regulator of the type III secretion system by directly binding to and activating its expression. RsmY and RsmZ antagonize this regulation by sequestering RsmA, thereby downregulating T3SS expression and modulating bacterial-host cell interactions (Marden et al., 2013; Intile et al., 2014).

It is well established that the Pseudomonas quinolone signal (PQS) not only functions as a quorum-sensing autoinducer but also directly modulates host immune responses. For instance, Kim et al. demonstrated that PQS and its precursor HHQ can downregulate innate immune responses through the NF-κB pathway, thereby dampening host inflammatory defenses (Kim et al., 2010). However, a critical knowledge gap remains: while PQS is known to exert immunomodulatory effects (Kushwaha et al., 2023), it is largely unexplored how *P. aeruginosa* dynamically regulates PQS synthesis via sRNA-mediated mechanisms to actively reshape the host immune microenvironment for the establishment of persistent colonization. In particular, whether there exists an sRNA that senses host immune status and coordinates bacterial virulence output with host immunomodulation through regulation of gene expression represents a fundamental unanswered question. Addressing this gap is of translational significance, as such an sRNA could serve as a dual-function target to simultaneously attenuate bacterial virulence and disrupt immune evasion.

Importantly, host pathogen interaction is dynamic. During early infection, the host mounts a pro-inflammatory response to eliminate bacteria (Copenhaver et al., 2015), yet *P. aeruginosa* can evade or redirect this response, for instance by inducing anti-inflammatory M2 macrophage polarization and suppressing pro inflammatory cytokines, to create a permissive niche for persistence (Sahu and Ruhal, 2025). Nevertheless, the molecular link between bacterial sRNAs and this immune reshaping process has been missing. Bridging this gap is translationally significant because targeting such sRNAs could simultaneously disrupt bacterial virulence and immune evasion, offering a new strategy against chronic infections where antibiotics fail.

In this study, using a mouse intraperitoneal infection model and RNA sequencing, we identified a novel sRNA, sRNA24, whose expression is dynamically regulated by host immune status. We demonstrate that sRNA24 does not affect bacterial growth but specifically promotes pyocyanin synthesis, biofilm formation, and host cell adhesion and invasion while inhibiting motility. Immunologically, sRNA24 suppresses M1 markers and pro-inflammatory cytokines but induces M2 markers and anti-inflammatory cytokines, thereby fostering an anti-inflammatory microenvironment. Mechanistically, sRNA24 functionally targets and upregulates pqsH, a key QS synthase gene. Overexpression of pqsH phenocopies sRNA24 effects. *In vivo*, the sRNA24 overexpressing strain enhances peritoneal colonization. Thus, sRNA24 connects QS, virulence, and host immune regulation, providing mechanistic insight into the potential adaptation of *P. aeruginosa* during persistent infection and suggesting a possible target for further investigation.

## 2 Materials and methods

### 2.1 Microbial strains and culture conditions

**Supplementary Table S1** presents the strains and plasmids. *E. coli* and *P. aeruginosa* PAO1 were cultured in Luria–Bertani medium at 37 °C. Antibiotics were incorporated where needed: ampicillin (100 μg/mL), gentamicin (25 μg/mL), and chloramphenicol (16 μg/mL).

### 2.2 Cell lines and experimental animals

RAW264.7 (15% fetal bovine serum(FBS) in high-glucose Dulbecco’s modified Eagle medium(DMEM) and A549 cells (10% FBS in high-glucose DMEM) were procured from Hunan Fenghui Biotechnology. C57BL/6J wild-type mice and NSG immunodeficient mice (6–8 weeks old, male) were obtained from Guangdong Yaokang Biotechnology Co., Ltd. All animal experiments were performed using C57BL/6J mice, except for the initial RNA-seq screening experiment, for which NSG mice were used to compare the effect of an intact versus compromised immune system on sRNA24 expression. All animal procedures were approved by the Laboratory Animal Ethics Committee of Qingyuan People’s Hospital (Approval No. LAEC-2024-001) and were conducted in accordance with the ARRIVE guidelines and the National Institutes of Health Guide for the Care and Use of Laboratory Animals. Mice were housed under specific pathogen-free conditions with a 12 h light/dark cycle and free access to food and water.

### 2.3 Transcriptome sequencing analysis

To identify small RNAs differentially expressed during infection, RNA-seq was performed on PAO1 bacteria under two conditions: *in vitro* culture in LB medium (control) and *in vivo* bacteria recovered from mouse peritoneal cavities. For the *in vivo* condition, C57BL/6J mice received an intraperitoneal injection of 200 μL PAO1 (2 × 10^6^ CFU). At 2 and 6 hours post-infection, mice were euthanized by CO_2_ inhalation, and peritoneal lavage fluid was immediately collected. The fluid was centrifuged, and the resulting bacterial pellet was subjected to total RNA extraction using TRIzol Reagent according to the manufacturer’s instructions. RNA concentration and purity were assessed using a Nanodrop spectrophotometer, and RNA integrity was confirmed by 1.5% agarose gel electrophoresis. Three independent biological replicates were prepared for each condition.

Qualified total RNA samples were submitted to Beijing Novogene Technology Co., Ltd. for library construction and sequencing. Paired-end sequencing was performed on the Illumina NovaSeq 6000 platform. Adapter sequences and low-quality reads were removed. The resulting clean reads were aligned to the *P. aeruginosa* PAO1 reference genome (NC_002516.2). Gene expression levels were quantified using featureCounts and normalized to CPM (Counts Per Million). Small RNAs were considered significantly differentially expressed if they met the criteria of |log_2_(fold change)| > 1 and an adjusted *P*-value < 0.05. Among the candidate sRNAs identified, sRNA24 was selected for subsequent functional studies due to its prominent differential expression level, high sequence conservation, and predicted targeting of QS-related genes.

### 2.4 Construction of gene overexpression strains

Following the method described by Lu et al. (2019), the coding sequences of sRNA24 and pqsH were cloned into the EcoRI site of pROp200 to generate pROp200-sRNA24 and pROp200-pqsH, respectively. The sRNA24 fragment was subsequently inserted into the PstI/HindIII sites of pSTV28 to construct pSTV28-sRNA24. For the reporter plasmid pUCP30T-pqsH-gfp, the target gene was inserted upstream of GFP in pUCP30T-gfp. All recombinant constructs were confirmed by DNA sequencing. **Supplementary Table S2** presents the primers used in this study.

### 2.5 Construction of gene knockout strains

Using the sacB-based allelic exchange system (Zeng et al., 2016), Briefly, upstream and downstream homology arms (each ~1000 bp) flanking the sRNA24 locus were PCR-amplified and cloned into the XbaI/SacI sites of the pGSM suicide vector, generating pGSM-ΔsRNA24. The construct was verified by Sanger sequencing and transformed into *E. coli* SM10λπ. Conjugation between *E. coli* SM10λπ and *P. aeruginosa* PAO1 was performed on LB agar at 37 °C for 6–8 h. Transconjugants were selected on Pseudomonas Isolation Agar containing gentamicin. To resolve the co-integrate, single colonies were grown overnight in LB without antibiotics, then plated on LB agar with 10% sucrose. Sucrose-resistant, gentamicin-sensitive colonies were selected, and deletion of sRNA24 was confirmed by PCR and Sanger sequencing. All primers are listed in **Supplementary Table S2**.

### 2.6 Real-time quantitative PCR

Total RNA was extracted using TRIzol, and cDNA was generated employing the ToloScript kit. Quantitative polymerase chain reaction (qPCR) was performed using 2 × Q3 SYBR qPCR Mix on a Bio-Rad CFX96 system. Relative expression was determined using the 2^−ΔΔCt^ method, with rpoD as the internal control for bacterial samples, mouse Gapdh for RAW264.7 cells, and human GAPDH for A549 cells.

### 2.7 Green fluorescent protein reporter assay

*E. coli* DH5α harboring pUCP30T-mRNA-gfp was co-transformed with pSTV28 or pSTV28-sRNA24. Cells were cultured, collected, and resuspended in PBS. OD_595_ and fluorescence (485 nm excitation/535 nm emission) were quantified utilizing a microplate reader. GFP activity was expressed as a fluorescence/OD ratio.Three independent biological replicates were performed.

### 2.8 Northern blot hybridization

Northern blot analysis was performed following the established protocol by Bhardwaj et al. (2020) Total RNA was extracted using TRIzol. Northern blotting was performed using biotin-labeled probes for sRNA24 and 5S rRNA to ascertain sRNA24 expression and transcript length.The experiment was repeated three times independently with similar results.

### 2.9 Growth curve determination

PAO1 cultures were standardized to an equivalent density, and 200 μL was dispensed to a 96-well plate. OD_600_ was continuously monitored at 37 °C with shaking, utilizing a microplate reader to generate growth curves. Each strain was tested in three biological replicates.

### 2.10 Quantitative analysis of pyocyanin

To 5 mL of bacterial culture, 3 mL of chloroform, and 1 mL of 0.2 N HCl were added and mixed. The absorbance of the chloroform layer was quantified at 520 and 600 nm. Pyocyanin concentration (μg/mL) was calculated as (A_520_/A_600_ × 17.072) (Essar et al., 1990). This assay was performed at least three times independently.The assay was performed with three independent biological replicates.

### 2.11 Biofilm formation assay

The assay was performed in 12-well plates. Each well received 2 mL of bacterial culture (normalized OD_600_) in LB and was incubated statically at 37 °C for 24 h. After removing planktonic cells and washing with PBS, adherent biofilm was stained with 1% crystal violet. The dye was dissolved in 95% ethanol, and OD_600_ was measured. Three biological replicates were performed.Each assay was performed in triplicate biological replicates.

### 2.12 Assessment of bacterial motility

For swimming assays, 5 μL of bacterial culture was spotted onto tryptone plates with 0.3% agar. For swarming, 5 μL was spotted onto LB plates containing 0.5% agar and 5 g/L glucose. Plates were incubated at 37 °C for 16 h, and the migration zone diameter was quantified.

### 2.13 Whole blood co-culture experiment

Human EDTA-anticoagulated whole blood was combined with PAO1 suspension at a 1:4 ratio and incubated with agitation at 37 °C for 6 h. Bacteria were pelleted via centrifugation, washed with PBS, and subjected to RNA extraction and qPCR.Three independent experiments were conducted using blood from different donors.

### 2.14 Cell infection assay

RAW264.7 macrophages and A549 epithelial cells were seeded into 6-well plates at a density of 5 × 10^5^ cells per well and cultured overnight to reach approximately 80% confluence. Prior to infection, the culture medium was replaced with serum-free DMEM. Bacterial strains grown to mid-log phase (OD_600_ ≈ 0.6) were washed twice with PBS and added to the cell monolayers at a multiplicity of infection (MOI) of 10:1. The plates were then incubated at 37 °C in 5% CO_2_ for 2 hours. Following infection, the medium was removed, and cells were gently washed three times with pre-warmed PBS to remove non-adherent extracellular bacteria. Total RNA was then extracted directly from the infected cell monolayers using TRIzol Reagent for subsequent qRT-PCR analysis of cytokine and polarization marker expression. Uninfected cells served as the baseline control group (NC). Each infection condition was tested in three independent biological replicates.Each infection condition was tested in three biological replicates.

### 2.15 Bacterial adhesion and invasion assay

The assay was performed as described by Edwards et al (Edwards and Massey, 2011). with minor modifications. A549 monolayers were infected with PAO1 at an MOI of 10:1 and incubated at 37 °C for 1 h to allow adhesion. After washing with PBS, cells were treated with DMEM containing 20 μg/mL polymyxin B for 2 h to kill extracellular bacteria (invasion assay). Cells were then lysed with 0.1% Triton X-100, and serial dilutions of the lysates were plated for CFU counting. Three independent biological replicates were performed.

### 2.16 Mouse intraperitoneal infection model

C57BL/6J mice received an intraperitoneal injection of 200 μL PAO1 (1 × 10^7^ CFU/mL) or PBS (control). Mice were randomly assigned to experimental groups (n = 3 per group per time point).The mice were euthanized at 2 and 6 h post-infection, using CO_2_ inhalation, and peritoneal lavage fluid was collected. The peritoneal lavage fluid contains a mixed cell population, and total RNA was extracted directly from the cell pellet obtained from the lavage fluid, which primarily consists of resident and recruited peritoneal cells, including macrophages, neutrophils, and lymphocytes. Following the method of Paul et al. (2024), an aliquot of the lavage fluid was serially diluted and inoculated onto LB agar plates for CFU enumeration, and another aliquot was utilized for RNA extraction and subsequent qRT-PCR analysis.All animal experiments were performed in compliance with the approved ethical protocol (see section 2).

### 2.17 Key bioinformatics software and databases

Target prediction: IntaRNA (https://rna.informatik.uni-freiburg.de/IntaRNA/Input.jsp), To predict potential targets of sRNA24, the sRNA24 sequence and the 5’-UTR regions of predicted operons across the entire PAO1 genome were used as query and target sequences, respectively. Prediction parameters were kept at default settings.

Sequence retrieval: NCBI database (https://www.ncbi.nlm.nih.gov/).

Secondary structure prediction: RNA Folding Form (https://www.unafold.org/mfold/applications/rna-folding-form.php).

Statistical analysis: GraphPad Prism 9.0, IBM SPSS Statistics 23.0.

### 2.18 Statistical analysis

All experiments were performed with at least three independent biological replicates. Technical replicates were performed for each biological replicate to ensure data reliability. Statistical analysis were based on biological replicates and are presented as mean ± standard error of the mean. Data normality was assessed by Shapiro-Wilk test before applying parametric tests. Differences between two groups were compared using Student’s *t*-test, and differences among multiple groups were compared using one-way analysis of variance (ANOVA), followed by Tukey’s *post-hoc* test for multiple comparisons. Non-normally distributed data were analyzed using Mann-Whitney U or Kruskal-Wallis tests. GraphPad Prism 9 software was utilized for all statistical analysis. Significance levels are indicated as: *, *P* < 0.05; **, *P* < 0.01; ***, *P* < 0.001; ****, *P* < 0.0001; ns, not significant.

## 3 Results

### 3.1 Screening and identification of sRNA24 and its expression characteristics during infection

We established a mouse intraperitoneal infection model (**Figure 1A**) to identify sRNAs playing key roles in infection. RNA-seq analysis revealed that sRNA24 may play a role in the interaction between infection and immunity. Notably, in immunocompetent C57BL/6J mice, sRNA24 expression progressively increased during infection, whereas in immunodeficient NSG mice it showed the opposite downward trend (**Figure 1B**). We successfully constructed an sRNA24 overexpression strain (24OE) and a knockout strain (Δ24) (**Figure 1C**) to validate this finding. Sequence analysis revealed that sRNA24 is an independent transcript of approximately 57 nt located between PA0906 and rsmA on the positive strand of the PAO1 genome (NC_002516.2, 991358-991414) (**Figure 1D**). The Northern blot confirmed that sRNA24 is a 57 nt non-coding short RNA (**Figure 1E**), and its secondary structure was predicted using RNA Folding Form (**Figure 1F**).To further validate its expression dynamics, we used two additional *in vitro* models. In co-culture with RAW264.7 macrophages, sRNA24 expression in PAO1 first decreased at 2 h and then increased at 6 h (**Figure 1G**). In a whole-blood infection model, sRNA24 expression initially decreased and later increased in the whole-blood group, whereas it showed an opposite trend in the heat-inactivated whole-blood group (**Figure 1H**). Collectively, these results indicate that sRNA24 expression is tuned by the host immune microenvironment, and its dynamics across different models are highly consistent with the initial sequencing findings.

**Figure 1:**
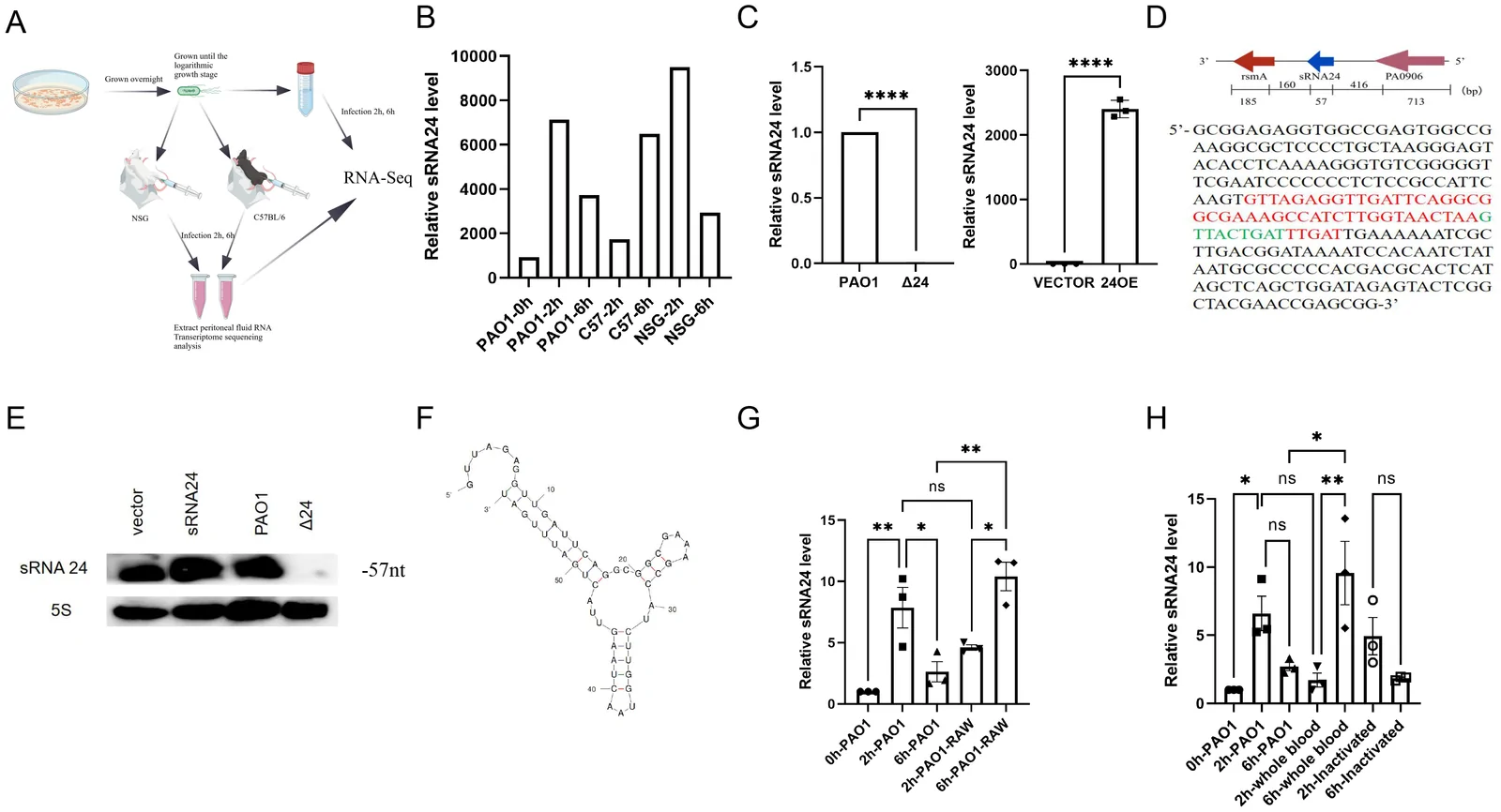
Identification of sRNA24 as an sRNA exhibiting an expression trend consistent with preliminary model data. **(A)** Schematic representation of the mouse intraperitoneal infection model. **(B)** Expression levels of sRNA24 in *in vitro* culture, C57BL/6J, and NSG mouse infection models (RNA-seq data). **(C)** qPCR validation of sRNA24 overexpression and knockout strain construction. **(D)** Genomic location of sRNA24 in PAO1: Top: Location of sRNA24 in the PAO1 genome, between PA0906 and rsmA; Red: rsmA; Blue: sRNA24; Purple: PA0906. Bottom: Sequence characteristics of sRNA24: Green highlight indicates the pqsH mRNA binding site; Red highlight indicates the sRNA24 sequence. **(E)** Northern blot validation of sRNA24. **(F)** Predicted secondary structure of sRNA24. **(G)** PAO1 co-culture model with RAW264.7 cells. **(H)** *In vitro* bloodstream infection model.RNA was collected at 2 and 6 hours post-infection to represent the early stage (acute inflammatory response phase, 2 h) and intermediate stage (immune cell recruitment and microenvironment remodeling phase, 6 h) of infection, respectively, allowing observation of the dynamic changes in sRNA expression. *P < 0.05; **P < 0.01; ****P < 0.0001; ns, not significant.

### 3.2 Regulation of *P. aeruginosa* virulence phenotypes by sRNA24

To examine the biological function of sRNA24, we initially assessed its impact on bacterial proliferation. The results revealed that neither knockout nor sRNA24 overexpression affected the basal growth rate of the bacteria (**Figure 2A**), indicating that subsequent phenotypic variations are unattributable to growth deficiencies. Subsequent phenotypic analysis revealed that sRNA24 positively modulates key virulence factors: sRNA24 enhanced pyocyanin production (**Figure 2B**) and improved biofilm formation (**Figure 2C**), whereas sRNA24 overexpression inhibited bacterial swimming and swarming abilities (**Figure 2D**) but enhanced host cell adhesion and invasion(**Figure 2E**). A549 alveolar epithelial cells were chosen for adhesion and invasion assays because they represent the first physical barrier that *P. aeruginosa* contacts and penetrates during respiratory infection. Although macrophages are central to innate immunity, their active phagocytosis would confound the assessment of bacterial adhesion to and invasion of epithelial cells. Thus, A549 cells were used to evaluate bacterial invasion of the epithelial barrier, whereas RAW264.7 macrophages were used to assess bacteria-phagocyte interactions and their immunomodulatory effects. The two models are complementary, reflecting host-pathogen interactions from distinct yet interconnected perspectives.

**Figure 2:**
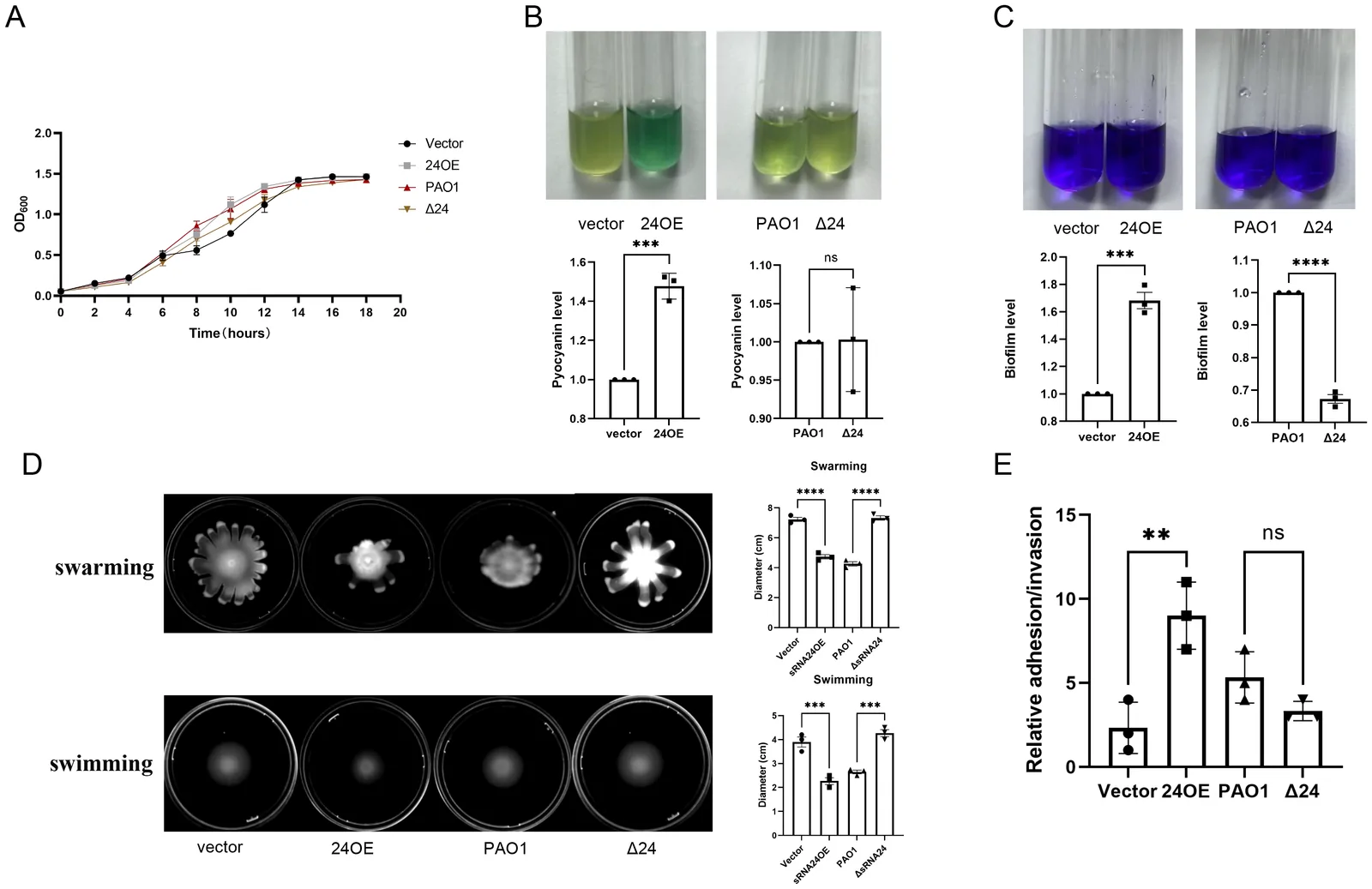
Effects of sRNA24 on virulence phenotypes. **(A)** Growth curves. **(B)** sRNA24 enhances pyocyanin production. **(C)** sRNA24 promotes biofilm formation. **(D)** sRNA24 inhibits swarming and swimming motility. Left panels show representative images, and right panels show quantitative analysis. **(E)** sRNA24 enhances adhesion/invasion ability (A549 cells). **P < 0.01; ***P < 0.001; ****P < 0.0001; ns, not significant.

At first glance, reduced motility might appear incompatible with enhanced adhesion and invasion. However, this apparent paradox reflects an adaptive transition from a motile, disseminating state to a sessile, colonizing state. Reduced motility favors bacterial retention at the infection site and promotes biofilm establishment, while enhanced adhesion and invasion likely reflect the activation of surface adherence mechanisms that are not strictly dependent on flagellum-driven motility. This phenotypic switch is consistent with the classic paradigm of *P. aeruginosa* shifting from acute to chronic infection. Thus, sRNA24 differentially modulates virulence traits, promoting properties associated with persistent colonization, including pyocyanin production, biofilm formation, and host cell adhesion and invasion, while suppressing those linked to early dissemination, such as motility.

### 3.3 sRNA24 promotes the formation of an anti-inflammatory immune environment

Co-culture with RAW264.7 macrophages showed that infection with the sRNA24 overexpression strain suppressed M1-type (pro-inflammatory) macrophage markers (CD80 and CD86) and promoted M2-type (anti-inflammatory) markers (Arg-1 and CD206) (**Figures 3A, B**). Cytokine analysis revealed that sRNA24 overexpression enhanced the anti-inflammatory cytokine Il-10 and inhibited the pro-inflammatory cytokine Tnf-α (**Figure 3B**). Consistent immunomodulatory effects were observed in co-culture with A549 alveolar epithelial cells (**Figure 3C**). These findings collectively demonstrate that sRNA24 actively reshapes the host immune response toward an anti-inflammatory profile.

**Figure 3:**
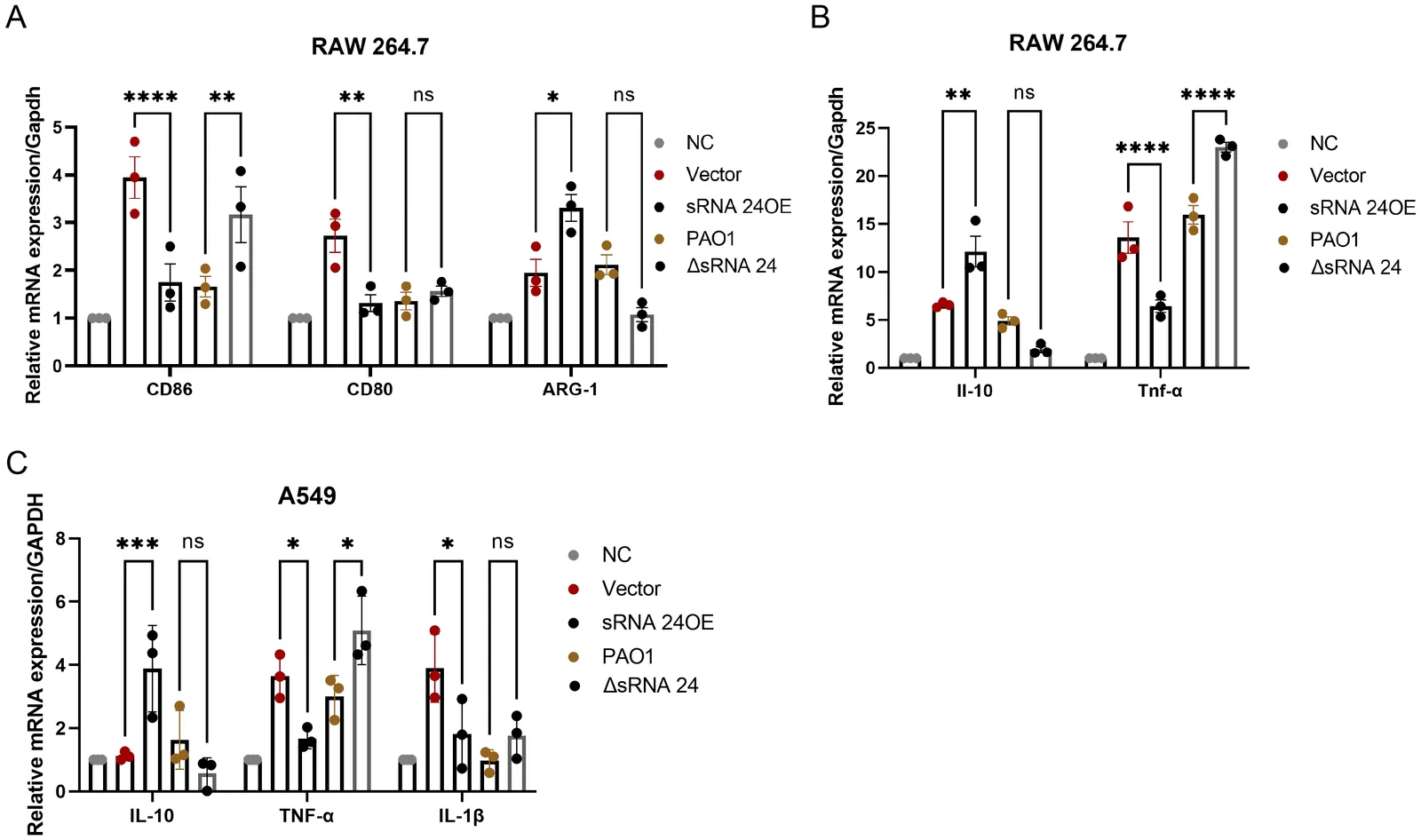
Effects of sRNA24 on cellular immune responses. **(A)** Effect of sRNA24 infection on cytokine expression in macrophages. **(B)** Effect of sRNA24 infection on macrophage polarization in RAW cells. **(C)** Effect of sRNA24 infection on cytokine expression in lung epithelial cells. *P < 0.05; **P < 0.01; ***P < 0.001; ns, not significant.

### 3.4 sRNA24 positively regulates the QS-related gene pqsH

To elucidate the molecular mechanism by which sRNA24 modulates virulence and host immunity, we used IntaRNA software to predict its potential targets. A particularly compelling candidate was pqsH, which encodes a key enzyme in PQS (Pseudomonas quinolone signal) synthesis, because PQS is known to regulate both virulence and immune responses. The prediction revealed a potential binding site between sRNA24 and the pqsH transcript (**Figure 4A**). To investigate whether sRNA24 functionally targets pqsH, we developed a green fluorescent protein (GFP) reporter system. (**Figure 4B**). The predicted sRNA24 target sequence from pqsH was inserted upstream of gfp to create pUCP30T-pqsH-gfp. This plasmid was co-transformed into *E. coli* DH5α together with the sRNA24 overexpression plasmid (pSTV28-sRNA24) or empty vector.The results showed that sRNA24 overexpression significantly enhanced GFP fluorescence intensity, whereas the control vector or mutation of the binding site abolished this effect (**Figure 4C**). This indicates that sRNA24 can functionally targets to pqsH mRNA. Moreover, qRT-PCR validated that sRNA24 overexpression in *P. aeruginosa* significantly increased pqsH transcription level (**Figure 4D**). Mechanistically, these findings reveal a novel regulatory axis: sRNA24 functionally targets pqsH mRNA and positively regulates its expression, thereby linking a sRNA to the core QS circuitry in a manner not previously described for this pathogen.

**Figure 4:**
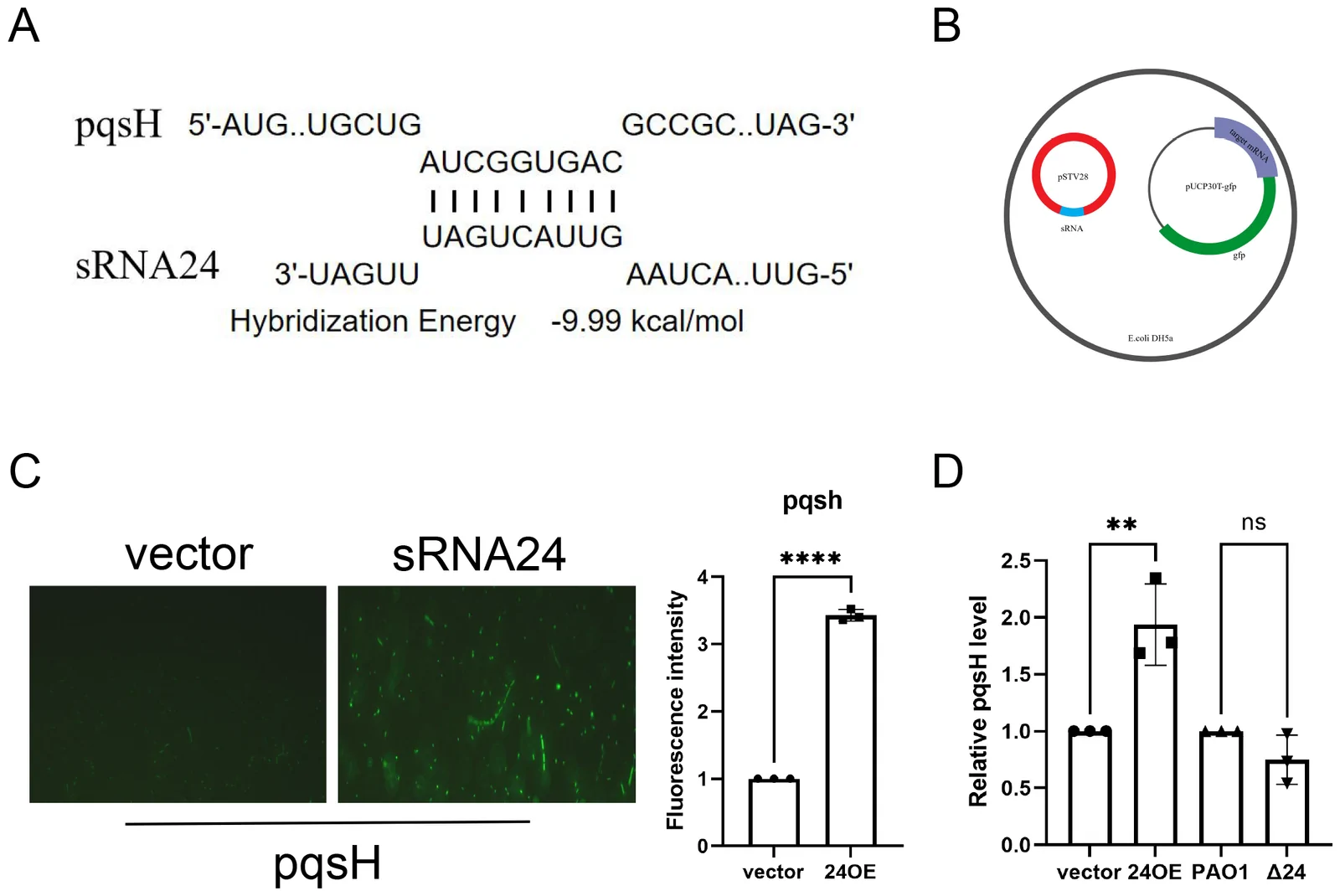
sRNA24 functionally targets the pqsH mRNA. **(A)** Predicted binding site between sRNA24 and pqsH as determined by the IntaRNA website. **(B)** Schematic representation of the GFP green fluorescent reporter system. **(C)** GFP fluorescence intensity detected by fluorescence microscopy (left) and microplate reader (right) after co-transformation of the pqsH-gfp reporter plasmid (which contains the predicted target sequence) into *E. coli* with the sRNA24 overexpression plasmid or its empty vector control. **(D)** Relative expression levels of pqsH in different strains detected by qRT-PCR. **P < 0.01; ****P < 0.0001; ns, not significant.

### 3.5 sRNA24 regulates bacterial virulence and host immunity via targeting pqsH

To determine whether the effects of sRNA24 are mediated through pqsH, we constructed a pqsH overexpression strain (**Figure 5A**). Phenotypic analysis revealed that pqsH overexpression significantly inhibited swarming and swimming motility (**Figure 5B**) and promoted pyocyanin production (**Figure 5C**) and biofilm formation (**Figure 5D**). Regarding immunomodulation, pqsH enhanced the synthesis of the anti-inflammatory cytokine Il-10 and suppressed the pro-inflammatory cytokine Tnf-α. Furthermore, pqsH downregulated the expression of M1 macrophage polarization markers CD80 and CD86 (**Figure 5E**). Consistent results were observed in A549 epithelial cells, where pqsH overexpression enhanced IL-10 production upon infection (**Figure 5F**). These data collectively demonstrate that pqsH is a key functional mediator of sRNA24-regulated virulence and immune modulation.

**Figure 5:**
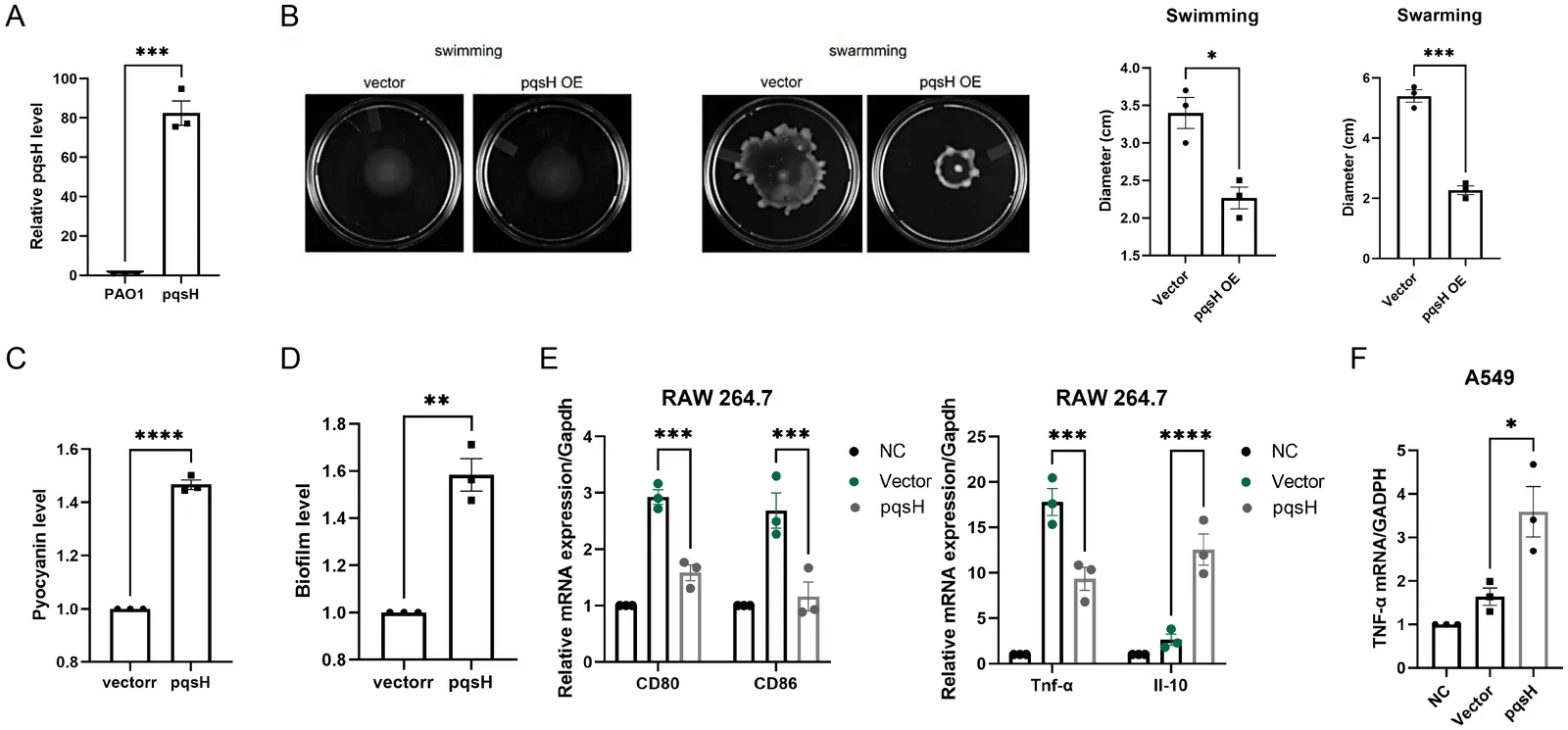
Regulation of virulence by the target pqsH and its mediated immunomodulation. **(A)** Construction of the pqsH overexpression strain. **(B)** pqsH inhibits swarming and swimming motility.Left panels show representative images, and right panels show quantitative analysis. **(C)** pqsH promotes pyocyanin production. **(D)** pqsH promotes biofilm formation. **(E)** pqsH promotes Il-10 production and inhibits Tnf-α production and suppresses the expression of M1 polarization markers CD80 and CD86. **(F)** pqsH promotes IL-10 production in A549 cells upon infection. ns, not significant; *P < 0.05; **P < 0.01; ***P < 0.001; ****P < 0.0001.

### 3.6 *In vivo* experiments confirm that sRNA24 enhances bacterial persistent infection capability by modulating the host immune microenvironment

In the mouse intraperitoneal infection model, the sRNA24 overexpression strain demonstrated significantly stronger colonization capacity post-infection (**Figure 6A**). Correspondingly, in the peritoneal fluid of mice infected with the sRNA24 overexpression strain, the mRNA expression level of the anti-inflammatory cytokine Il-10 was significantly increased, whereas the mRNA levels of the pro-inflammatory cytokines Tnf-α and Il-6 were significantly suppressed (**Figure 6B**). These *in vivo* findings suggest that sRNA24 may modulate the host immune microenvironment toward an anti-inflammatory phenotype, potentially contributing to early colonization and a tendency toward persistence.

**Figure 6:**
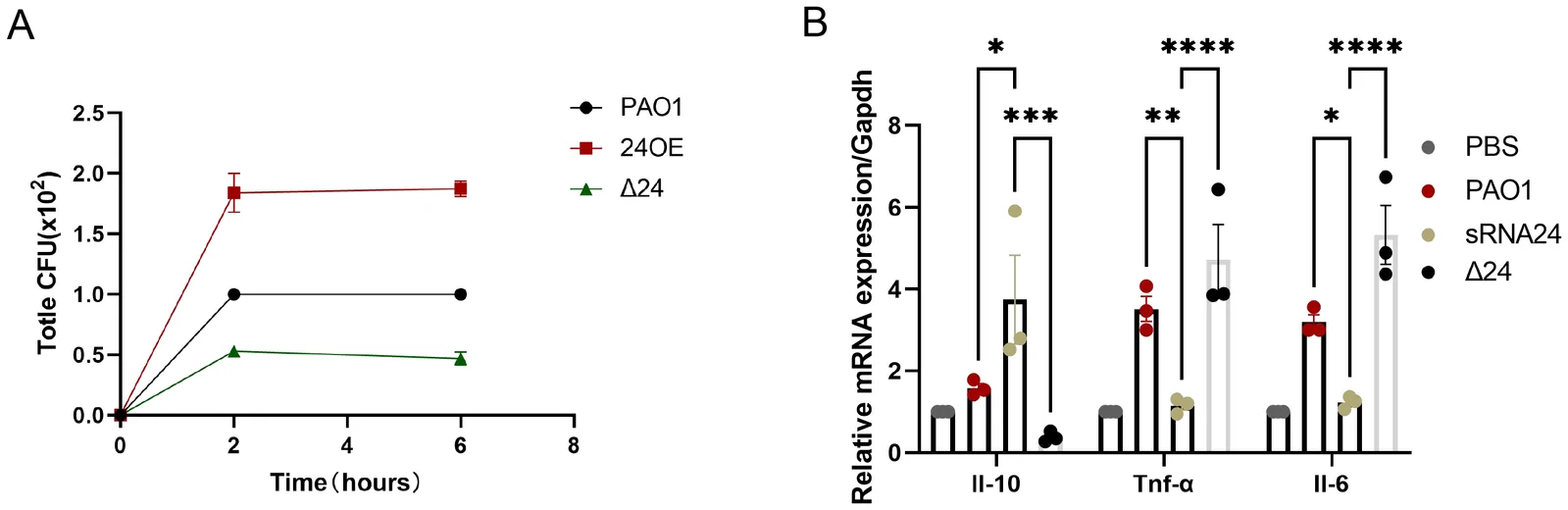
*In vivo* colonization and immunomodulatory effects of sRNA24 in a mouse intraperitoneal infection model. **(A)** CFU counts in mouse peritoneal fluid at 2 and 6 h post-infection. **(B)** qRT-PCR detection of the effect of sRNA24 on the expression levels of inflammatory cytokines in mouse peritoneal fluid. All experiments in this figure were performed using male C57BL/6J mice (6–8 weeks old). *P < 0.05; ***P < 0.001; ns, not significant.

## 4 Discussion

The therapeutic challenge of *P. aeruginosa* infection stems from its complex virulence regulatory network and robust immune evasion capabilities. Comprehending the molecular mechanisms by which bacteria detect the host environment and synchronize virulence expression with immune remodeling is essential for formulating novel anti-infective strategies. This study isolated and characterized a novel sRNA, sRNA24, whose expression is dynamically regulated by the host immunological microenvironment. We further demonstrated that sRNA24 coordinates bacterial virulence phenotypes and modulates the expression of inflammatory mediators at the transcriptional level by functionally targeting pqsH, suggesting a potential immunomodulatory mechanism, a key gene in the quorum sensing system. This provides a previously unrecognized mechanism by which *P. aeruginosa* dynamically adapts to the host environment and may establish persistent colonization. sRNA24 creates favorable conditions for early colonization and a tendency toward persistence.

The gradual upregulation of sRNA24 expression in the immunocompetent mouse infection model, in contrast to its downregulation in immunodeficient NSG mice, indicates that its expression is modulated by the host immunological condition. *In vitro* co-culture models further validated this phenomenon: in macrophage co-culture and *in vitro* bloodstream infection models, sRNA24 initially decreased and subsequently increased, whereas this dynamic disappeared in the heat-inactivated whole blood group. This signifies that sRNA24 expression is finely regulated by active components within the host immune microenvironment. Similar to other sRNAs that respond to host signals (*Salmonella* PinT, *P. aeruginosa* PrrF1/F2) (Reinhart et al., 2017; Felden and Augagneur, 2021), the dynamic expression of sRNA24 presumably indicates stage specific adaptation of the bacterium during infection. Downregulation in the early phase may enhance motility and invasion, while upregulation in the subsequent phase helps remodel the immune microenvironment, facilitate biofilm formation, and establish persistent infection.

sRNA24 demonstrates differential regulation of virulence factors: promoting pyocyanin synthesis and biofilm formation, inhibiting swimming/swarming motility, and improving adhesion/invasion to host cells. This strategy of promoting colonization and inhibiting dissemination is beneficial during the initial phase of infection, where bacteria inhibit motility to evade immune clearance, and enhance biofilm and adhesion for stable colonization; virulence factors such as pyocyanin can directly damage host cells. Similar regulatory patterns are observed in other bacterial sRNAs, including *S. aureus* RNAIII, which is involved in biofilm regulation (Kim et al., 2017) and *P. aeruginosa* RsmY/Z mediating the switch from acute to chronic infection (Janssen et al., 2018). This indicates that sRNA24 may play a role in coordinating complex virulence output.

sRNA24 infection significantly inhibited the expression of M1-type macrophage polarization markers (CD80 and CD86) and the pro-inflammatory cytokine TNF-α, and promoted M2-type markers and the secretion of the anti-inflammatory cytokine IL-10. *In vivo* experiments further validated this effect: Il-10 levels were markedly increased, whereas Tnf-α and Il-6 were suppressed in the peritoneal fluid of mice infected with the sRNA24 overexpression strain, concomitant with enhanced bacterial colonization. These consistent *in vitro* and *in vivo* findings indicate that sRNA24 may contribute to early colonization and a tendency toward persistence by promoting an anti-inflammatory immune microenvironment. This provides initial evidence for a potential immune-modulatory mechanism initiated by a bacterial sRNA.We further explored the potential molecular mechanisms linking the sRNA24-pqsH axis to macrophage polarization. It has been reported that PQS and HHQ can downregulate innate immune responses through the NF-κB pathway (Kim et al., 2010). Our data suggest that sRNA24 upregulates pqsH to promote PQS synthesis, which may suppress pro-inflammatory responses via the NF-κB pathway. Additionally, PQS has been shown to act as a ligand for the aryl hydrocarbon receptor (AhR), potentially influencing immune cell differentiation (Jia et al., 2025). We speculate that the sRNA24-pqsH axis may also affect macrophage polarization through PQS-induced oxidative stress.

This study demonstrated that sRNA24 can functionally target the pqsH mRNA and positively modulate its expression. pqsH encodes a key enzyme in PQS synthesis, and the PQS system is an integral component of the *P. aeruginosa* QS network, regulating multiple virulence factors and immunomodulation (Kim et al., 2010; Lin et al., 2018; Miranda et al., 2022). Comparing sRNA24 with other regulatory sRNAs in *P. aeruginosa* reveals both similarities and functional diversity within the sRNA regulatory network. PhrS activates PqsR to upregulate PQS synthesis and pyocyanin production under hypoxic conditions (Sonnleitner et al., 2011) PrrF1/F2 modulate PQS synthase genes under iron limitation (Oglesby et al., 2008), enhancing virulence factor output. RsmY/Z sequester RsmA to mediate the switch from acute to chronic infection (Heurlier et al., 2004). Notably, the recently reported PqsS sRNA targets pqsL to indirectly increase PQS production and promote swarming motility (Jia et al., 2024), thereby facilitating bacterial dissemination and acute infection establishment. In contrast, sRNA24 directly upregulates pqsH, enhances biofilm formation, inhibits motility, and favors persistent colonization. This functional disparity between sRNA24 and PqsS may stem from targeting different nodes of the same pathway: sRNA24 directly enhances PQS synthesis through pqsH upregulation, whereas PqsS indirectly increases PQS production by reducing metabolic consumption through the HQNO branch pathway (Heurlier et al., 2004). This comparison suggests that *P. aeruginosa* may employ multiple sRNAs to synergistically or antagonistically modulate different nodes of the same pathway, enabling fine-tuned virulence output at different infection stages.

By overexpressing pqsH, we successfully recapitulated the principal phenotypic changes induced by sRNA24 overexpression, indicating that pqsH overexpression is sufficient to recapitulate the major phenotypic effects of sRNA24 overexpression, strongly suggesting that pqsH is a key downstream effector of sRNA24 function. However, epistasis experiments are needed to definitively establish its necessity.

Based on their regulatory role in acute/chronic infection, known sRNAs can be initially categorized as those that facilitate acute infection and inhibit chronic infection [PrrF1/2 (Djapgne et al., 2018), PqsS (Jia et al., 2024)]; those promoting chronic infection and inhibiting acute infection [RsmY/Z (Janssen et al., 2018)]. By promoting biofilm formation, inhibiting motility, and enhancing adhesion, sRNA24 exhibits traits associated with persistent colonization, presenting a regulatory mode that may balance persistent colonization with virulence output, thereby aiding bacteria in maintaining a survival advantage during prolonged coexistence.

The present study has several limitations. First, our *in vivo* experiments were performed using an intraperitoneal infection model rather than the more clinically relevant pulmonary infection model, and this model choice represents a limitation. While the intraperitoneal model allows for convenient bacterial recovery and rapid assessment of colonization capacity, it does not fully recapitulate the complex anatomical and physiological features of respiratory infections, such as the airway epithelial barrier, mucociliary clearance, and alveolar microenvironment (Vintrych et al., 2022; Rodgers et al., 2023). Therefore, our *in vivo* findings should be considered as preliminary proof-of-concept, and validation in a pulmonary infection model is essential to confirm the translational relevance of the sRNA24-pqsH regulatory axis. Second, the conclusions regarding cytokine regulation and macrophage polarization are based primarily on mRNA expression levels, lacking systematic protein-level validation (e.g., ELISA, flow cytometry). Third, epistasis experiments (ΔpqsH combined with sRNA24 overexpression) to definitively establish the necessity of pqsH in mediating sRNA24 effects have not yet been completed. Fourth, bacterial colonization was evaluated only at 2 and 6 hours post-infection, a time window that does not support definitive conclusions regarding chronic infection. Fifth, chromosomal complementation strains for deletion mutants were not constructed. Sixth, we acknowledge that this study only assessed total biofilm biomass by crystal violet semi-quantification and did not perform in-depth analysis of biofilm three-dimensional structure or extracellular matrix components, which is a limitation of this study. These limitations will be addressed in future studies, including the construction of complementation strains and validation in pulmonary infection models.

This study identified sRNA24 in *P. aeruginosa* whose expression is dynamically modulated by the host immunological microenvironment. sRNA24 targets and positively regulates pqsH expression, thereby activating the PQS quorum-sensing system, which differentially regulates bacterial virulence phenotypes and modulates host inflammatory responses at the transcriptional level, correlating with enhanced bacterial colonization *in vivo*. In summary, we identify a novel signaling pathway regulated by sRNA24 that connects the QS system, virulence expression, and host immunological modulation (**Figure 7**), presenting a potential target for further investigation in the context of persistent infections caused by this bacterium.

**Figure 7:**
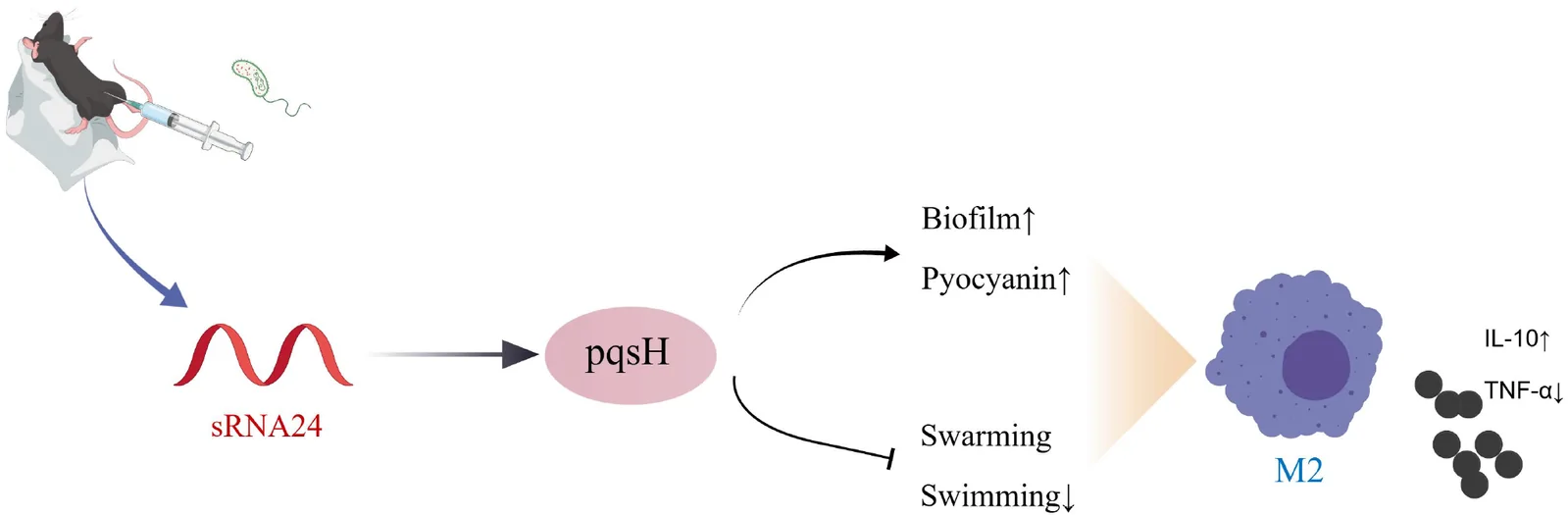
Regulatory model of the sRNA24-pqsH axis in *P. aeruginosa* infection and immune evasion.

## Data Availability

The data presented in the study are deposited in the OMIX, China National Center for Bioinformation/Beijing Institute of Genomics, Chinese Academy of Sciences (OMIX) repository, accession number OMIX018289.
