# Correction: Regulatory role and mechanism of sRNA24 targeting *pqsH* in *Pseudomonas aeruginosa* infection immunity

**DOI:** 10.3389/fcimb.2026.2027194

**Published:** 2026-10-21

**Authors:** Huanlan Liao, Qianying Liu, Yuyang Liu, Qiwei Li, Yujuan Xiong, Lan Wang, Yang Lu

**Affiliations:** 1 Department of Laboratory Medicine, The Second Affiliated Hospital of Guangzhou University of Chinese Medicine, Guangzhou, Guangdong, China; 2 Guangzhou University of Chinese Medicine, Guangzhou, Guangdong, China; 3 Department of Laboratory Medicine, Panyu Hospital of Guangzhou University of Chinese Medicine, Guangzhou, Guangdong, China; 4 Department of Laboratory Medicine, Chengdu Integrated TCM & Western Medicine Hospital, Chengdu, Sichuan, China; 5 Department of Clinical Laboratory, The Affiliated Qingyuan Hospital of Guangzhou Medical University (Qingyuan People’s Hospital), Qingyuan, Guangdong, China

**Keywords:** immune evasion, pqsH, *Pseudomonas aeruginosa*, quorum sensing, small RNA, virulence regulation

In the published article, in the **Funding** Statement, an incorrect number was provided for the “Guangdong Basic and Applied Basic Research Foundation”. The correct number is “2023A1515220103”.

The original version of this article has been updated.

