# Supplementary material for: Regulatory role and mechanism of sRNA24 targeting pqsH in Pseudomonas aeruginosa infection immunity
Source: Front Cell Infect Microbiol. 2026 Oct 8;16:1933779. doi: 10.3389/fcimb.2026.1933779 (PMC13732399; doi:10.3389/fcimb.2026.1933779)
Supplement: Supplementary file 1 [file SupplementaryFile1.docx]

# Regulatory role and mechanism of sRNA24 targeting pqsH in *Pseudomonas aeruginosa* infection immunity

**Huanlan Liao^1^,** **Qianying Liu^1^, Yuyang Liu^2^, Qiwei Li^1^, Yujuan Xiong^1#^, Lan Wang^1, 3#^, Yang Lu^1, 3#^**

**Table S1.** Information of primers used in this study

| Primer | Sequence (5’ to 3’) | Purpose |
| --- | --- | --- |
| In28-*sRNA24*-F | TCCTCTAGAGTCGACCTGCAGGTTAGAGGTTGATTCAGG | pSTV28-*sRNA106* |
| In28-*sRNA24*-R | ACGACGGCCAGTGCCAAGCTTATCAAATCAGTAACTTAGTTACC |  |
| In200-*sRNA24*-F | atcggctcgtataatgaattcGTTAGAGGTTGATTCAGG | pROp200-*sRNA106* |
| In200-*sRNA24*-R | cgaattttaacaaaagaattcATCAAATCAGTAACTTAGTTACC |  |
| 24-P1 | ACGGCCAGTGAATTCGAGCTCATCGCCTTCAACCGCGAC | ΔsRNA24构建 |
| 24-P2 | ACTTGAATGGCGGAGAGGGGATTTTATCCGTCAAGCGATTTTTTCA |  |
| 24-P3 | TGAAAAAATCGCTTGACGGATAAAATCCCCTCTCCGCCATTCAAGT |  |
| 24-P4 | GGCTGGATCCCAAGCTCTAGAAGCTTCGTCGCGCAGATC |  |
| *sRNA24*-F | GTTAGAGGTTGATTCAGGC | qRT-PCR |
| *sRNA24*-R | ATCAAATCAGTAACTTAGTT |  |
| *pqsH*-F | ATGACCGTTCTTATCCAGGG | qRT-PCR |
| *pqsH*-R | CTACTGTGCGGCCATCTCAC |  |
| gfp-*pqsH*-F | AGTTCTAGAATGACCGTTCTTATCCAGGG | pUCP30T-*pqsH*-*gfp* |
| gfp-*pqsH*-R | AGTCCATGGCTACTGTGCGGCCATCTCAC |  |
| 200-*pqsH*-F | atcggctcgtataatgaattcATGACCGTTCTTATCCAGGG | pROp200-*pqsH* |
| 200-*pqsH*-R | cgaattttaacaaaagaattcCTACTGTGCGGCCATCTCAC |  |
| *rpod*-F | CTGAAGATCGCCAAAGAGCC | qRT-PCR |
| *rpod*-R | GTGTGGTCGGTGTTCATGTC |  |
| GAPDH-F | AGGAGCGAGACCCCACTAACA |  |
| GAPDH-R | AGGGGGGCTAAGCAGTTG |  |
| IL-6-F | CTTGGGACTGATGCTGGTGACA |  |
| IL-6-R | GCCTCCGACTTGTGAAGTGGTA |  |
| IL-1β-F | GCACTACAGGCTCCGAGATGAA |  |
| IL-1β-R | GTCGTTGCTTGGTTCTCCTTGT |  |
| TNF-α-F | GCACCACCATCAAGGACTCA |  |
| TNF-α-R | AAGAGGAGGCAACAAGGTAGAG |  |
| m-TNF-ɑ-F | TACTGAACTTCGGGGTGATCG |  |
| m-TNF-ɑ-R | GGGTCTGGGCCATAGAACTGA |  |
| m-CD206-F | TACACAAATTCAGGGTTCTGG |  |
| m-CD206-R | GATGCTGCTGTTATGTCTCTG |  |
| m-Arg1-F | GAACTGAAAGGAAAGTTCCCA |  |
| m-Arg1-R | AATGTACACGATGTCTTTGGC |  |
| m-CD80-F | TCAAGTTTCCATGTCCAAGG |  |
| m-CD80-R | GTTCATCAACATCTGAAGACAC |  |
| m-CD86-F | CTGATCTCAGATGCTGTTTCC |  |
| m-Cd86-R | AGCCTTTGTAAATGGGCAC |  |

F: forward primer, R: reverse primer.

**Table S2.** Information of Probe sequence used in this study

| Probe | Sequence (5’ to 3’) | 5’ modification |
| --- | --- | --- |
| sRNA24 | GCTTTCGCCGCCTGAATCAACCTCTAA | Biotin |
| 5S | GUUUCACUUCUGAGUUCGGCAUGGGAUCAGGUGGG | Biotin |
